# A field deployable method for a rapid screening analysis of inorganic arsenic in seaweed

**DOI:** 10.1007/s00604-017-2151-1

**Published:** 2017-03-18

**Authors:** Edi Bralatei, Karolina Nekrosiute, Jenny Ronan, Andrea Raab, Evin McGovern, Dagmar B. Stengel, Eva M. Krupp, Joerg Feldmann

**Affiliations:** 1 TESLA (Trace Element Speciation Laboratory), Department of Chemistry, University of Aberdeen, Aberdeen, Scotland AB24 3UE UK; 2 Marine Institute, Rinville, Oranmore, Co. Galway Ireland; 3 Botany and Plant Science, School of Natural Sciences, Ryan Institute for Environmental, Marine and Energy Research, National University of Ireland Galway, Galway, Ireland

**Keywords:** Gutzeit method, Speciation, Hyphenated method, HPLC-ICPMS, Seaweed, Laminaria, Kelp

## Abstract

Inorganic arsenic (iAs) in 13 store-bought edible seaweed samples and 34 dried kelp (*Laminaria digitata*) samples was determined by a newly developed, field-deployable method (FDM) with the aid of a field test kit for arsenic in water. Results from the FDM were compared to results from speciation analysis achieved by using high performance liquid chromatography coupled to inductively coupled plasma mass spectrometry (HPLC-ICP-MS). The FDM consisted of a simple extraction method using diluted HNO_3_ to quantitatively extract iAs without decomposing the organoarsenicals to iAs followed by the selective volatilisation of iAs as arsine (AsH_3_) and subsequent chemo-trapping on a filter paper soaked in mercury bromide (HgBr_2_) solution. Method optimization with a sub-set of samples showed 80–94% iAs recovery with the FDM with no matrix effect from organo-arsenic species in the form of dimethylarsinic acid (DMA) on the iAs concentration. The method displayed good reproducibility with an average error of ±19% and validation by HPLC-ICP-MS showed that the results from the FDM were comparable (slope = 1.03, R^2^ = 0.70) to those from speciation analysis with no bias. The FDM can be conducted within an hour and the observed limit of quantification was around 0.05 mg kg^−1^ (dry weight). This method is well suited for on-site monitoring of iAs in seaweed before it is harvested and can thus be recommended for use as a screening method for iAs in seaweed.

**Electronic supplementary material:**

The online version of this article (doi:10.1007/s00604-017-2151-1) contains supplementary material, which is available to authorized users.

**Graphical abstract:**
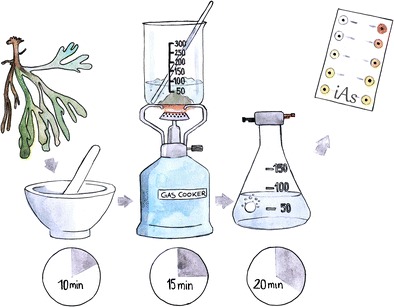
Screening seaweed for their inorganic arsenic concentration within one hour without bias has been made possible in the field by using a field deployable arsenic kit. Its accuracy and precision was compared to HPLC-ICPMS.

## Introduction

Seaweeds are widely used in a range of industrial applications, ranging from (production of hydrocolloids, to uses in health, agri/horticulture, cosmetics and as human and animal feed) [1–5]. An increased awareness of their potential high nutritional values due to high vitamin, essential minerals, fibre, proteins and carbohydrate content has led to their use as a food source for humans and animals [1, 6]. Commonly used for food are Nori (*Pyropia tenera*, *Pyropia yezoensis*; Rhodophyta) Kombu (*Saccharina japonica*), Wakame (*Undaria pinnatifida*), Hijiki or Hizikia (*Hizikia fusiforme*) (all Phaeophyceae) and Sea Lettuce (*Ulva* spp.; Chlorophyceae) [7, 8]. Not only is it common for sheep to graze on seaweeds in coastal regions of Scotland [9, 10], Iceland and some other European countries, seaweeds also serve as animal feed and as additives to improve animal diet [11, 12]. Commonly used seaweeds for additives in land animal feed and countries where they are used include; *Ascophyllum nodosum* (Norway and United Kingdom (UK)), and *Laminaria digitata* (France) where they are used in poultry, pig, ruminant and rabbit diet; both are also commonly used in Iceland [13].

Due to their versatile use, cultivation of seaweeds has become a common practice in over 35 countries around the world with Asian countries as highest producers [12]. However, studies have shown the presence of high levels of As, a known class 1 carcinogen when in the inorganic forms (arsenite (As^III^) and arsenate (As^V^)) [14, 15] in different seaweed species. Seaweeds are thought to absorb As as As^V^ through phosphate channels due to its similarities to phosphate (H_2_PO_4_^−^ versus H_2_AsO_4_^−^) [16]. After absorption, As^V^ is transformed to arsenosugars which make up 80–90% of the total As in most seaweed species [17].

In June 2015, The FAO/WHO Joint Committee set maximum contaminant limits for iAs in commercial food products like rice and rice based products with high levels of iAs [18]. But so far, no maximum contaminant limit is set for iAs in seaweed and seaweed based products used for food or animal feed [19]. Instead, countries like France, the first country to regulate iAs in seaweed and the United States have set a regulatory level of ≤3 mg kg^−1^ in seaweed approved for food [20]. However, in February 2014, the UK Food Standard Agency stated in its food safety policy update that a maximum contaminant level for As in seaweed is no longer considered necessary as the issue only applies to Hijiki rather, consumer advice is appropriate [21]. Hijiki seaweed is known to contain high level of iAs (50–60% of total As present as iAs) [22]. This high level of iAs is suggested to be due to a lack of genetic capability of As detoxification. However, Taylor and Jackson have shown in 2016, considerable but highly variable levels of iAs (2.8–20.4 mg kg^−1^) present in *Laminaria digitata* depending on the region of cultivation on the Atlantic East coast of the USA [16] and recently, we were able to confirm also high levels of iAs present in *Laminaria digitata* from Ireland (Ronan et al., in preparation)*.*

However, there is still a lack of data for the occurrence of iAs and its variability in seaweeds. This may be due to the fact that, the analytical methods for determining iAs in seaweed are complicated and in most cases, hyphenated techniques such as the coupling of HPLC to an ICP-MS are required. These methods are well established analytical methods with detection limits in the sub part per billion (ppb) levels but, they involve the collection and transportation of samples to laboratories from their natural habitat. Commonly used is anion exchange column chromatography coupled online to ICP-MS as arsenic-specific detector. The As species are identified by retention time and if structural information of novel compounds are needed the simultaneous analysis with ESI-MS/MS (Electrospray Ionization Mass Spectrometry) can also be applied [23]. Other methods applied include HPLC-HG-AFS (HPLC coupled with Hydride Generation Atomic Fluorescence Spectrometry), ICP-AES (Inductively Coupled Plasma Atomic Emission Spectrometry) and capillary electrophoresis coupled with ICP-MS [24–26]. The use of these methods may be established but it has been shown in a proficiency test that even expert laboratories did not agree on the level of iAs in seaweed [27]. The reason given was possible co-elution with other organo-arsenicals. To avoid this ambiguity, post-column hydride generation with HPLC-HG-ICP-MS can be applied to separate the hydride-forming species from other arsenicals [28]. The volatilisation reaction of the iAs to AsH_3_ is highly species-specific. With subsequent gas-liquid separation after hydride generation, iAs can be separated and detected unequivocally from organo-arsenicals under strongly acidic conditions. It has even been shown that hydride generation by itself is selective enough when directly coupled to ICP-MS for detection of iAs only [29]. But these above methods are all laboratory-based involving high running costs, long analysis time and data processing requiring skilled and trained personnel. The running conditions and bulkiness of these tools make them unsuitable for field analysis. As a result of the continuous distribution of toxic As species in the environment and the vast application and consumption of seaweeds, a low cost, easy to use but field deployable method (FDM) is necessary for iAs determination in seaweed which can determine if the seaweed harvested contains iAs above or below a regulatory limit.

We were able to show that a field method based on the Gutzeit reaction (Eqs. 1 and 2) [30] was capable of accurately determining concentration of iAs in rice in the field with the aid of a field kit [31].

1 $$ {2\mathrm{H}}_3{\mathrm{AsO}}_4+{2\mathrm{H}}_3{\mathrm{O}}^{+}+{2\mathrm{Na}\mathrm{BH}}_4\to {2\mathrm{AsH}}_3+2\mathrm{B}{\left(\mathrm{OH}\right)}_3+{4\mathrm{H}}_2\mathrm{O}+{2\mathrm{Na}}^{+} $$

2 $$ {\mathrm{AsH}}_3+{\mathrm{H}\mathrm{gBr}}_2\to {\mathrm{H}}_2\mathrm{As}-\mathrm{HgBr}+\mathrm{HBr} $$

The field kit used in this study comprises of easily transportable reagents (sulfamic acid and sodium borohydride), lead acetate impregnated filters for H_2_S removal and HgBr_2_ impregnated filter paper for AsH_3_ collection. The reaction involves the reduction of iAs present in samples to the corresponding volatile species (AsH_3_) using sulfamic acid and sodium borohydride. The volatile species is then trapped on the HgBr_2_ impregnated filter paper giving a coloured spot depending on the iAs concentration. The coloured spot is compared with a pre-determined colour chart to get a concentration range or inserted in a digital photometric reader for concentration readout in μg L^−1^. In the previous study [31], iAs was selectively determined with the field method without contribution from DMA, the most common organic species present in rice. In this study we show the application of an optimized field method for the determination of iAs in seaweed which contains mainly arsenosugars and arsenolipids.

## Experimental section

### Chemicals and reagents

Arsenic standards were prepared from the corresponding salts. Double distilled water (< 18 MΩ cm) was used for the preparation of reagents and standards. Disodium methylarsenate (MMA) and sodium arsenate were from ChemService (West Chester, USA (www.chemservice.com)), Cacodylic acid (DMA) was from Strem Chemicals (Newburyport, USA (www.strem.com)). Sodium Borohydride (99%) was from Acros Organics (Geel, Belgium (www.acros.com)), Rhodium used for internal standard was from Specpure (Alfa Aesar, Germany (www.alfa.com)), and Ammonium carbonate was from BDH UK (www.vwr.com). Sodium borohydride tablets and sulfamic acid were from Palintest UK (www.palintest.com).

### Samples

A total of 47 samples were analysed. 13 commercially available edible seaweeds (3 Kombu, 4 Nori, 3 Wakame and 3 Hijiki – no scientific names provided on the commercial packaging, originating from Japan, China and Korea) were purchased online and from local shops and supermarkets around Aberdeen, UK (Table S1). Thirty four *Laminaria digitata* samples related to different parts of the seaweed from their natural habitat in Ireland. More details on the samples were reported elsewhere (Ronan et al., in preparation), since this study is focussing only on the use of a FDM for screening of iAs in seaweed in general.

### Sample storage

Air-dried seaweed samples were milled with a coffee grinder to achieve homogeneity and particle size below 0.1 mm, stored in sealed plastic bags at room temperature.

### Quality control and statistical analysis

For quality assurance seaweed reference material ERM-CD200 (*Fucus vesiculosus*) was used for total arsenic measurement. For totals and speciation analysis ERM-BCR-211, a rice flour with certified total As and iAs was analysed alongside the samples. For significance tests, the two-way student’s *t*-test was used unless otherwise stated.

### Optimisation of field method

100 mg of milled Hijiki seaweed was extracted with 1%, 3%, 5% and 10% HNO_3_ by boiling for 15–20 min and cooled in a water bath for 15 min. Samples were extracted in triplicates and analysed for total iAs (sum of As^III^ and As^V^) using a commercially available As field test kit for water analysis (Wagtech Digital Arsenator (Palintest, UK)).

To check the effect of sample matrix on the concentration of iAs detected with the field method, sub-samples of Nori, Wakame, Kombu and Hijiki were spiked with known concentration of arsenite (5, 10, 50 and 100 μg L^−1^) after sample extraction with 5% HNO_3._ This was done to check if the sample matrix has any effect on the recovery of iAs present in sample using the FDM.

#### Sample preparation for speciation analysis by HPLC-ICP-MS

For speciation analysis, the supernatants of the extracts were centrifuged at 3000 rpm after cooling, 100 mL of H_2_O_2_ was added to samples to oxidize As^III^ to As^V^ before analysis.

#### Sample preparation for total As analysis by ICP-MS

To determine the total As in the seaweed samples, 200 mg of sample was digested in 2 mL HNO_3_ (70%) and 1 mL H_2_O_2_ (30%). Microwave digestion was carried out on the CEM Mars 5 system (CEM Microwave Technology Ltd., U.K.). The digestion program is as follows; a 5 min ramp to 50 °C, hold for 5 min, followed by 5 min ramp to 75 °C and hold for another 5 min, and finally hold at 95 °C for 30 min.

#### Inorganic arsenic determination with the field method

The Wagtech digital Arsenator (Palintest UK Ltd) was used for the determination of iAs in seaweed extracts. Extracted sample was emptied into reaction flask without centrifuging, 2 to 3 drops of antifoam B emulsion (Sigma-Aldrich, Missouri, USA) was added followed by a sachet of sulfamic acid and a tablet of NaBH_4_. The reaction flask was sealed with a tri-filter bung device holding As collecting filter paper impregnated with HgBr_2_ for trapping AsH_3_, a scrubber for excess As removal and lead acetate filter for H_2_S removal. Sample was left standing for 20 min to allow the reaction come to completion after which the As collection filter paper was taken out and compared to a pre-determined colour chart or inserted in the digital detector for a readout in μg L^−1^. The actual iAs concentration in the seaweeds is calculated based on the amount of samples extracted.

#### Arsenic speciation using HPLC-ICP-MS

The Agilent 1100 HPLC and Agilent 7500c ICP-MS system were used for separation and detection of As species. Species separation was done using a gradient program (Table S1). A Hamilton PRP-X100 anion exchange column (dimensions: 10 μm, 4.1 × 250 mm) with 200 mM carbonate buffer (pH 9.2) as the mobile phase (flow rate: 1 mL min^−1^) and a sample injection volume of 100 μL was used. Rhodium (Rh) was used as the internal standard which was added to the sample stream between the column and the nebulizer via a T-piece, and mass to charge (m/z) ratios As 75; Se 77, 82; and Rh 103 were selected for detection. DMA standards (5, 10, 50, 150, 100 μg L^−1^) were used for system calibration, peaks were integrated with Origin 61 software and were quantified accordingly. Although a gradient program was employed, no change in arsenic response was observed.

#### Total arsenic determination in seaweed

The ICP-MS (Agilent 7500c series) was used for the determination of total As present in seaweed samples. The analysis was carried out in reaction cell mode with hydrogen as reaction gas and argon gas as the carrier gas. System was calibrated with As standards in 1% HNO_3_ (5, 10, 50, 150 μg L^−1^), rhodium was used as the internal standard and mass to charge ratios As 75; Se 77, 82; and Rh 103 were selected for detection.

## Results and discussion

### Field method optimisation

**Fig. 1:**
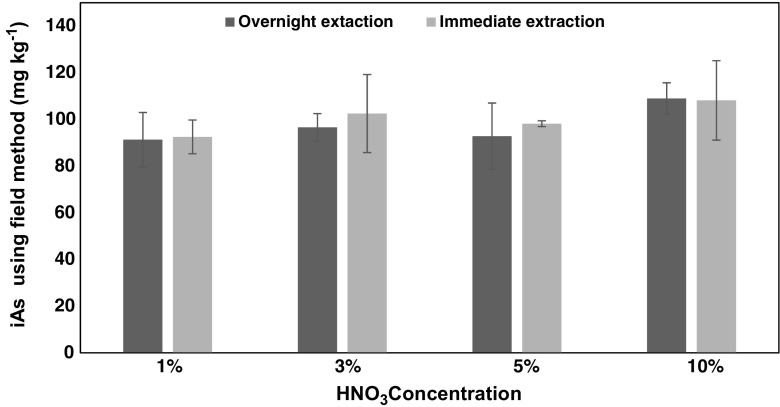
Effect of immediate and overnight extraction on iAs concentration detected by the field method using a sample of Hijiki. The error bars reflect the standard deviation for *n* = 3. All pairs are not significantly different (paired *t*-test *p* > 0.05) while increased HNO_3_ show also no significant increase (two-way *t*-test *p* > 0.05)

To determine the optimal extraction condition, different nitric acid concentrations (1%, 3%, 5% and 10%) were investigated using Hijiki sample (H2). Samples were heated immediately after adding the acid solutions or left standing overnight before heating. There was no significant difference (*P* = 0.17) in the concentration of iAs detected with immediate extraction or overnight extraction with different acid concentrations (Fig. 1). Hence, iAs seemed to be extracted immediately which is useful if this FDM is used for a fast on-site screening method.

**Fig. 2:**
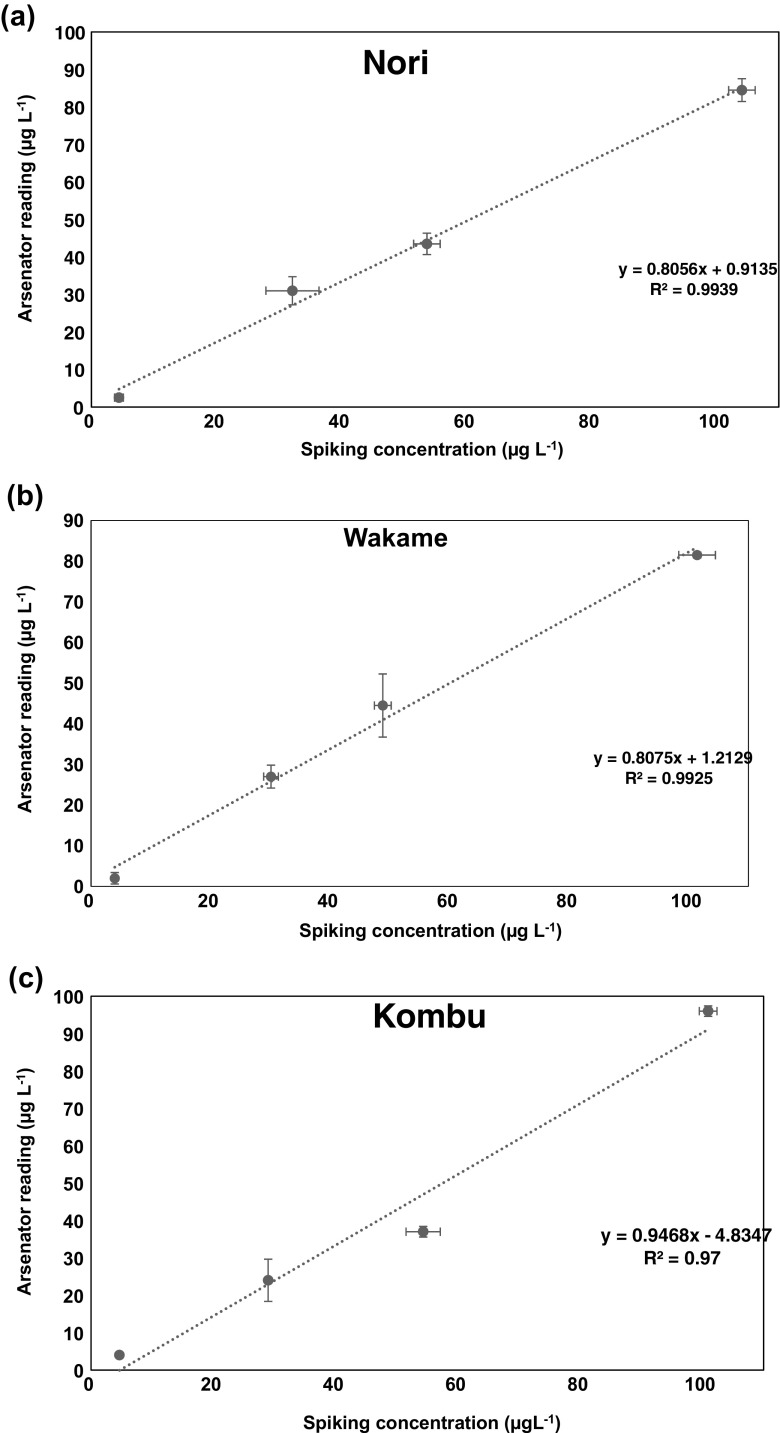
a-c Improved iAs recovery in Nori (80%), Wakame (80%) and Kombu (94%) after sample dilution with water to alleviate matrix effect. % recoveries for the samples were inferred from the slopes

Since the use of a centrifuge is not feasible in field like conditions, the sample were analysed without centrifuging. Hence, the effect of sample matrix on the iAs concentration detected was also tested. One sample each from the four types of commercial seaweed samples (Nori, Wakame, Kombu and Hijiki) were spiked with increasing concentration of iAs (5, 30, 50 and 100 μg L^−1^ As^III^) after extraction. While the results show linearity between the spiking concentration and the detected concentration, recovery for iAs was between 69% and 72% except Hijiki which gave a recovery of 86% (identified in the slope of the spike in supplementary information Fig. S1-S4). The high iAs recovery for Hijiki can be attributed to the amount of sample used during extraction and further dilution before analysis (100 mg of Hijiki and 5 g for other samples). While diluting the Hijiki samples after extraction was intended to reduce the iAs concentration in the samples to a quantifiable concentration, this also helped in alleviating the matrix effect on the iAs recovery with the FDM. To improve the iAs recovery in Nori, Wakame and Kombu sub-samples, samples were left standing for 30 mins after extraction to separate the residue from the supernatant (without centrifugation). The supernatant was decanted and diluted with double distilled water (~1:1) before analysis on the field kit. Improved recovery (80–94%) was observed for all 3 sub-samples (Fig. 2a **–c**).

**Fig. 3:**
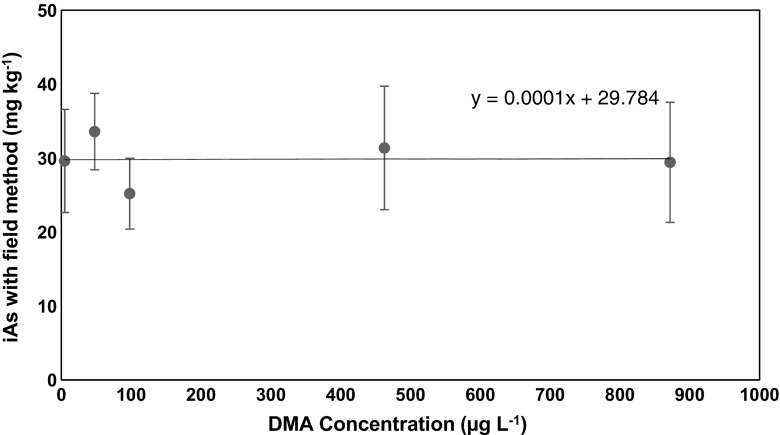
Effect of DMA concentration present in sample (H1) on the concentration of iAs concentration detected by the field method. No noticeable influence of DMA concentration on iAs detected

Furthermore, due to the occurrence of different As species in seaweeds [32], the contribution of organo-arsenicals to the concentration of iAs detected by the field method was also investigated. Results show no significant increase in the concentration of iAs detected with 10% HNO_3_ extraction compared to the lower concentrations of HNO_3_ used for extraction (deduced from Fig. 1). This indicates that a possible decomposition of organo-arsenic species during extraction with 10% HNO_3_ did not contribute to the concentration of inorganic arsenic detected by the field kit. For further confirmation, a Hijiki sample (H1) was spiked with increasing concentration of DMA, a major metabolite and a likely product of possible acid degradation of arsenosugars and arsenolipids [33, 34]. The iAs detected after spiking and extraction of sample was independent of the amount of DMA present as confirmed by Fig. 3.

### Quality control

For quality control of total As and iAs results, reference material ERM BCR-211 (rice flour) was analysed alongside the samples and ERM-CD200, a seaweed CRM with a certified value for total As. ERM-CD200 gave a value of 53 ± 2.6 mg kg^−1^ for total As which was also not significantly different from the certified value of 55 ± 4 mg kg^−1^. Total As and iAs results for ERM-BCR-211 were 0.22 ± 0.016 mg kg^−1^ and 0.11 mg kg^−1^ which were not significantly different from the certified values of 0.26 ± 0.013 mg kg^−1^ and 0.124 ± 0.011 mg kg^−1^. This confirms that the values for iAs obtained by HPLC-ICP-MS for the samples were accurate. Hence, this method is used as a reference method for validating the FDM. Due to the high amount of sample used during extraction for the FDM, no reference material was analysed along with the samples. Furthermore, the available reference material for seaweed only has a certified value for total As and not iAs. Hence this could not be analysed along with sample using the FDM.

### Determination of iAs in edible seaweed samples using the FDM

Thirteen commercial seaweed samples belonging to red and brown macroalgae were analysed with the field method. Samples were also analysed with HPLC-ICP-MS for iAs in order to check if the FDM is capable of identifying seaweed samples with high levels of iAs. The total As concentrations of these samples varied according to the type of seaweed from 19 to 235 mg kg^−1^ (Table S3). The lowest concentration for total was much higher than the regulatory limit of 3 mg kg^−1^ set for iAs (Regulatory limit in France and the United States) hence further testing of samples for iAs was required. iAs was first determined by HPLC-ICP-MS following the optimized extraction method as part of the FDM. To identify iAs present in samples based on the retention time on the separation column, samples were spiked with arsenate and compared with non-spiked samples (supplementary information Fig. S4). Since the primary aim of this study was testing the accurate determination of iAs in various seaweed by the FDM with the aid of the field kit, the other As peaks (i.e. peaks 1 and 2 presumably DMA or arsenosugars in Fig. S5) present in the samples were not quantified. As expected the Hijiki samples had extremely high concentration of iAs (34–75 mg kg^−1^ with HPLC-ICP-MS) while all other edible seaweed samples showed concentrations below 1 mg kg^−1^ (Table S3). The sample set seemed useful for testing the capability of the field method hence all samples were analysed in triplicates. 9 out of the 13 samples gave readings above the l.o.q on the field kit given as 0.05 mg kg^−1^(dry weight). This value is similar to what has been established for rice in the previous field study [31]. Furthermore, variable concentrations of iAs was observed for the different algae and as confirmed from the speciation analysis, Hijiki samples had the highest iAs concentration (53 ± 7–76 ± 20 mg kg^−1^). Reproducibility of +/− 18% was observed in these samples for *n* = 3 (Table S3) when analysed with the field method. The lowest iAs concentrations were observed in Wakame (0.05 ± 0.02–0.06 ± 0.02 mg kg^−1^) and Nori (0.06 ± 0.06–0.3 ± 0.01 mg kg^−1^), with concentrations close to or below the l.o.q. of the field kit (Table S3).

### Application of FDM to kelp related samples

To further assess the field method in terms of accuracy, 34 samples related to different parts of *L. digitata* collected from western Ireland were analysed. Speciation analysis by HPLC-ICP-MS of these samples from Ireland showed elevated and variable iAs concentrations (Ronan et al., in preparation). The concentration range in the samples was between 2.2–87 mg kg^−1^ with an average of 29 ± 19 mg kg ^−1^. The results from the study are in accord with a study on iAs in kelp samples from the Atlantic coast in the NE of the US [16]. More discussion on the implication for the use of kelp as feed or food was discussed elsewhere (Ronan et al., in preparation).

Analysis of the kelp samples with the FDM gave concentrations between 3 and 73 mg kg^−1^ and an average concentration of 34 ± 18 mg kg^−1^. Average iAs concentration from the FDM was compared to that of speciation analysis by ICP-MS (29 ± 19 mg kg^−1^) and no significant difference was established using a paired student’s *t*-test (*p* = 0.217). The method average reproducibility for the analysed samples at *n* = 3 was similar to the value established for Hijiki samples (± 19%). The established error is acceptable taking into consideration the variable iAs concentration present in the samples. The individual absolute error in general was between ±10 mg kg^−1^ with some outliers up to 40 mg kg^−1^. These high values however are not a result of extremely high concentration of total As. The correlation between the absolute error and the amount of total or organo-arsenicals in the seaweed could not be established (Fig. S6).

**Fig. 4:**
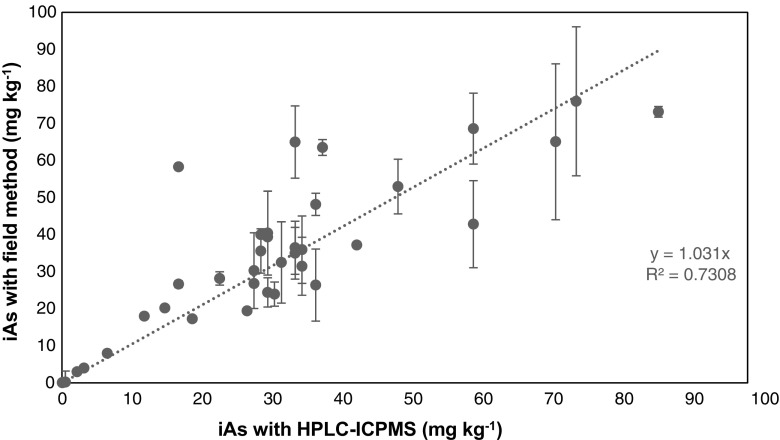
Linear regression showing the relationship between iAs concentration in seaweed samples using the FDM and HPLC-ICP-MS. Error bars are given as the standard deviation of triplicate (*n* = 3)

To further validate the results obtained from the FDM, a linear regression for all measured seaweed samples (edible seaweed and kelp) between the FDM and the HPLC-ICP-MS showed that the results for iAs obtained from the FDM were comparable to data from speciation analysis by HPLC-ICP-MS (Fig. 4). The linear regression graph gave a slope = 1.03 and an R^2^ of 0.70 which indicates a correlation between both methods with very limited bias. This is more encouraging than the results from the spiking experiments. Considering the lower concentration of iAs in the samples at around the legislation of 3 mg kg^−1^, no false positive or false negative would have been recorded using this field deployable method.

While it is important to warn consumers against the presence of toxins in food products, the continuous monitoring and setting of regulatory limits for toxic contaminants in food and livestock feed is very important since it is an important step for minimising exposure to these contaminants.

Taking into consideration the reported high levels of iAs in some seaweed species used as food and the increasing practice of seaweed cultivation, a regulatory procedure is important. This is deemed necessary due to possible exposure to toxicologically relevant As species through seaweed consumption.

Most analytical procedures for As determination can involve chromatographically separating As species or selectively generating their corresponding volatile hydrides before detection with ICP-MS, AAS or AFS (see Table 1). Recent studies show improved analysis time after sample extraction of these methods (faster separation and detection) [35–39] and can also offer detection limits down to the sub ppb level but their use in the field or field like conditions is next to impossible. The FDM described in this body of work can serve as a screening tool for determining iAs in the field before processing seaweed for food, animal feed or fertilizer production.

**Table 1:** Recently reported rapid test methods for determining iAs in food, their different assay time and limits of detection

Method	Analysis time* (min)	Limit of detection (μg kg^−1^)	References
HG-ICP-MS	4	5	Petursdottir et al. [35]
IC-ICP-MS/MS	4.5	0.15	Jackson [36]
HPLC-ICP-MS	3	0.01	Narukawa *et al.* [37]
Field kit (Gutzeit reaction)	20	5	Bralatei *et al.* [31] and this study
SPE-AFS	-	1.1	Huang *et al.* [38]
SPE-HG-AAS	6	20	Rasmussen *et al.* [39]

*Represents time taken for sample analysis after sample extraction. (−) no specific time stated

## Conclusion

Most laboratory-based techniques are more than adequate to accurately and precisely determine iAs in different seaweed species. However, they are commonly expensive, time–consuming and require highly trained analysts. This study has shown that a newly developed, field deployable method can be used to (precisely within the error of +/−19%) determine iAs in seaweed samples with 80–95% recovery. The results from the FDM, which were achieved within one hour, were comparable to those obtained by HPLC-ICP-MS analysis with limited bias, and were applicable to a vast range of iAs concentration in the samples.

So far, no legislative limits have been set by the FAO/WHO joint committee for iAs in seaweed used for human consumption and animal feed. The iAs concentration in some of the samples analysed varied more than 2 orders of magnitude more than the regulatory limit (3 mg kg^−1^) set by France and the United States [20]. This FDM was able to determine which samples were above or below this limit. This is a further indication that the method can be used potentially for on-site screening of iAs in seaweed in the field and also serve as a tool for monitoring iAs in seaweed in situ, as well as routine screening by regulatory bodies.

## Electronic supplementary material

ESM 1 (PDF 434 kb)
