# Supplementary material for: A field deployable method for a rapid screening analysis of inorganic arsenic in seaweed
Source: Mikrochim Acta. 2017 Mar 18;184(6):1701–9. doi: 10.1007/s00604-017-2151-1 (PMC5429896; doi:10.1007/s00604-017-2151-1)
Supplement: Supplementary file 1 — (PDF 434 kb) [file 604_2017_2151_MOESM1_ESM.pdf]

Supplementary Material

A field deployable method for a rapid screening analysis of inorganic arsenic in seaweed

Edi Bralatei[1], Karolina Nekrosiute[1], Jenny Ronan[2], Andrea Raab[1], Evin McGovern[2], Dagmar B. Stengel[3], Eva M. Krupp[1], Joerg Feldmann[1,*]

[1] TESLA (Trace Element Speciation Laboratory, Department of Chemistry, University of Aberdeen, Aberdeen, AB24 3UE, Scotland, UK

[2] Marine Institute, Rinville, Oranmore, Co. Galway, Ireland

[3] Botany and Plant Science, School of Natural Sciences, Ryan Institute for Environmental, Marine and Energy Research, National University of Ireland Galway, Galway, Ireland

Table S1 Sample details for edible seaweed samples bought in stores around Aberdeen and online.

Table S2 Gradient setting for As species separation on the Hamilton PRP-X100 with 200 mM ammonium carbonate buffer,

Table S3 Total As, iAs and percentage iAs in edible seaweed samples determined by ICP-MS, the FDM and HPLC-ICP-MS.

Figure S1-4: Effect of sample matrix on iAs recovery after spiking Hijiki, Nori, Kombu and Wakame samples with increasing concentration of As^{III} using the field method. Percentage iAs recovery for each sample was inferred from the slope of the linear equation.

Figure S5: Chromatogram of Hijiki sample (H2) showing the retention time for iAs in sample and sample spiked with As^{V}. Spiked sample was used to identify the retention time As^{V}. Peaks 1 and 2 (presumably arsenosugars) were not quantified since the aim of the study was to test the accurate determination of iAs with the field method

Figure S6: Relative error of iAs versus the concentration of organo-arsenicals in seaweed samples show no correlation. Although there were a few outliers which is not as a result of the amount organo-arsenicals, the relative error for most of the individual samples is approximately ± 50%

Figure S7 Absolute error of iAs versus the concentration of organo-arsenicals present in samples show no correlation.

Table S1 Commercial seaweed sample information

Seaweed type	Sample ID	Country of origin	Supplier name	Shop
Kombu	K1	China	Fuzhou Hailin Food Co.Ltd	Mathew's shop, Aberdeen
Kombu	K2	China	Jiasheng	Mathew's shop, Aberdeen
Kombu	K3	Korea	Wel.Pac	Online
Nori	N1	China	Nagai's	Mathew's shop, Aberdeen
Nori	N2	China	Yutaka	Morrisons, Aberdeen
Nori	N3	China	Takaokaya	Mathew's shop, Aberdeen
Nori	N4	Unknown	Yamamotoyamai	Mathew's shop, Aberdeen
Wakame	W1	Japan	Clearspring	Online
Wakame	W2	China	Yutaka	Online
Wakame	W3	Japan	Wel.Pac	Online
Hijiki	H1	Japan	Clearspring	Online
Hijiki	H2	South Korea	Fujiko	Online
Hijiki	H3	Korea	Wel.Pac	Online

Table S2 Gradient setting for species separation on the Hamilton PRP-X100 with 200 mM ammonium carbonate buffer

Time (min)	% Buffer
2	0.1
15	5.0
30	25.0
35	25.0
35.01	0.1

Table S3 Total As, iAs and percentage iAs in edible seaweed samples determined by ICP-MS, the FDM and HPLC-ICP-MS (error is given as the standard deviation of triplicates)

Sample ID	Total As (mg kg^{-1})	iAs FDM (mg kg^{-1})	iAs HPLC-ICP-MS (mg kg^{-1})	% iAs from total As (FDM)
K1	19 ± 0.8	0.07 ± 0.05	0.12	0.4
K2	62 ± 2.6	0.12 ± 0.04	0.09	0.2
K3	115 ± 6.7	n.m.[c]	n.m.	n.m.
H1	160 ± 36	65 ± 9.76	34	40.6
H2	234 ± 0.9	76 ± 20	75	32.5
H3	135 ± 5.5	53 ± 7.4	49	39.3
W1	48 ± 5.4	0.05 ± 0.02	< l.o.q.[b]	0.1
W2	69 ± 0.6	0.06 ± 0.02	n.m.	0.09
W3	76 ± 2.1	< l.o.q.[a]	0.02	n.m.
N1	43 ± 1.2	< l.o.q.	< l.o.q.	n.m.
N2	34 ± 1.3	0.25 ± 0.01	n.m	0.7
N3	37 ± 1.1	0.06 ± 0.06	0.52	0.2
N4	38 ± 1.8	< l.o.q.	< l.o.q.	n.m.

[a]l.o.q (limit of quantification) for field method 0.05 mg kg^{-1}; [b] l.o.q for HPLC-ICPMS: 0.01 mg kg^{-1} [c]n.m. (not measured)

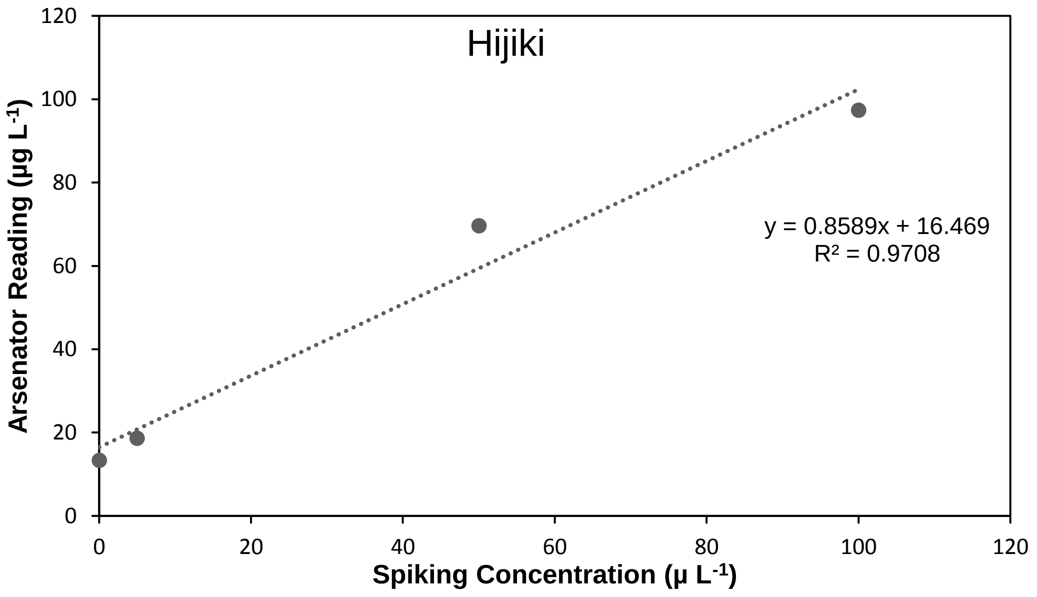

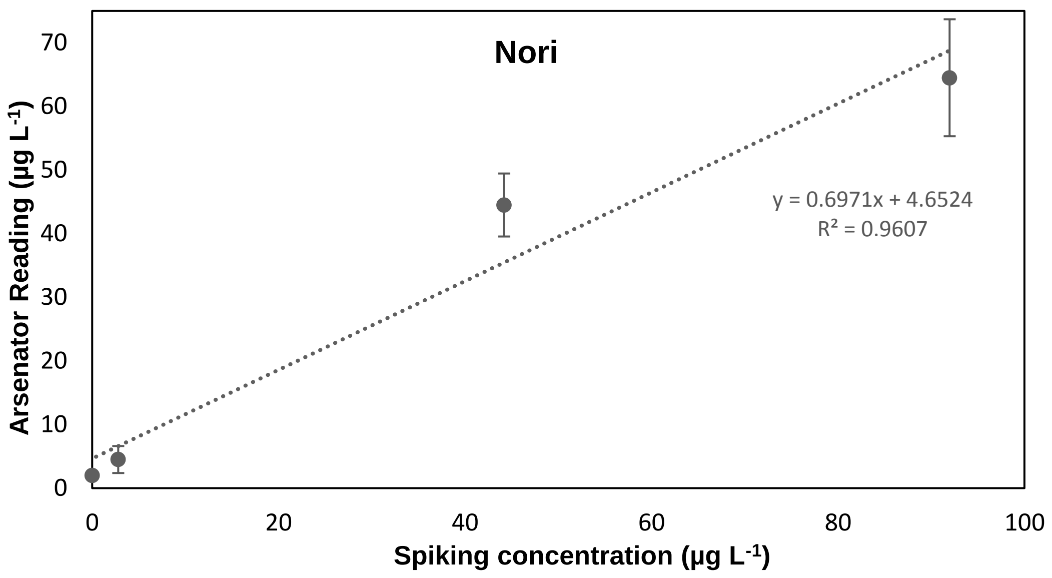
Nori
y = 0.6971x + 4.6524
R² = 0.9607
Arsenator Reading (µg L-1)
Spiking concentration (µg L-1)
0
10
20
30
40
50
60
70
0
20
40
60
80
100

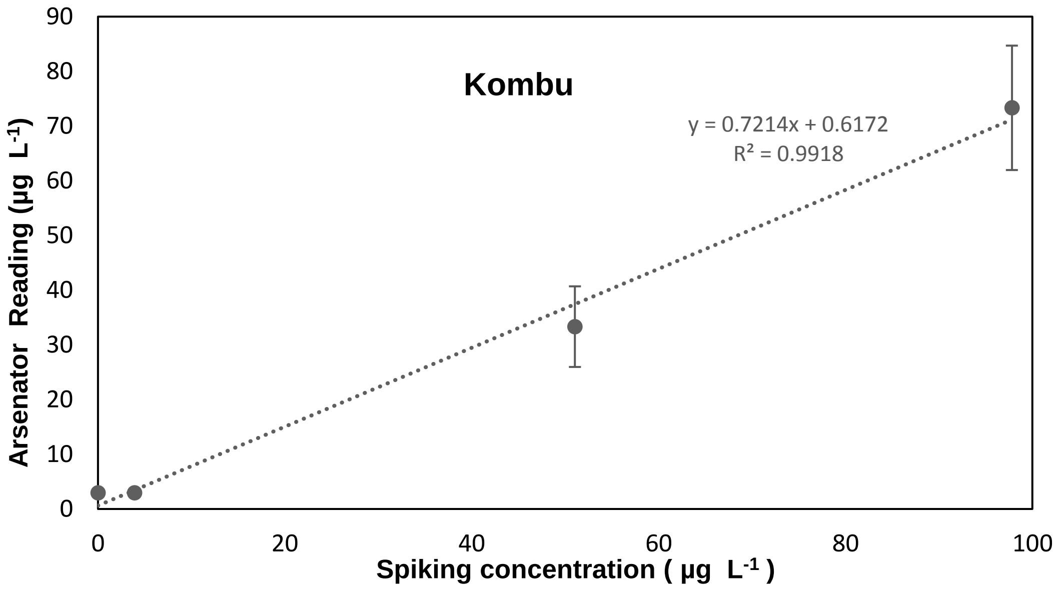
Kombu
y = 0.7214x + 0.6172
R² = 0.9918
Arsenator Reading (µg L-1)
Spiking concentration (µg L-1)
0
10
20
30
40
50
60
70
80
90
0
20
40
60
80
100

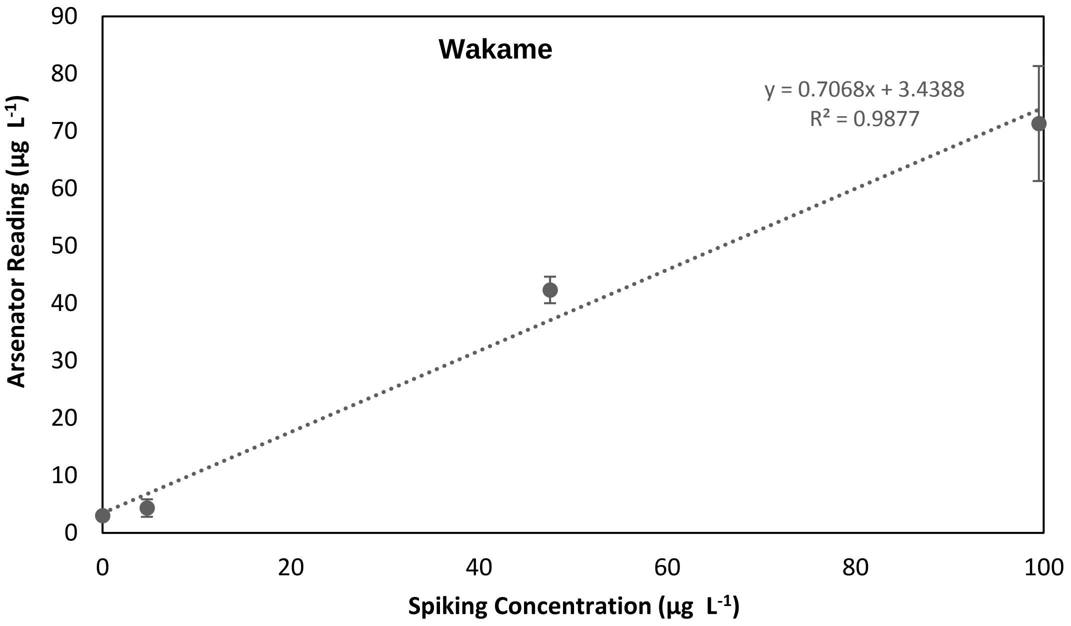

Figure S1-4 Effect of sample matrix on iAs recovery with the field method after spiking Hijiki, Nori, Kombu and Wakame samples with increasing concentration of As^{III} (5, 10, 50 and 100 µg L^{-1}).

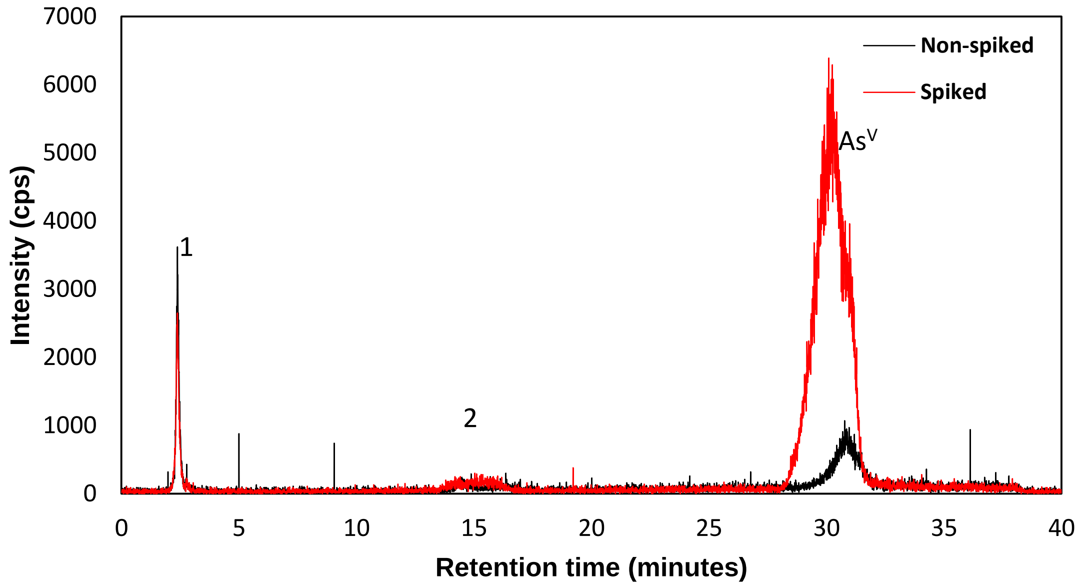

Figure S5: Chromatogram of Hijiki sample (H2) showing the retention time for iAs in sample and sample spiked with As^{V}. 1 and 2 are arsenosugars and their organoarsenic degradation products.

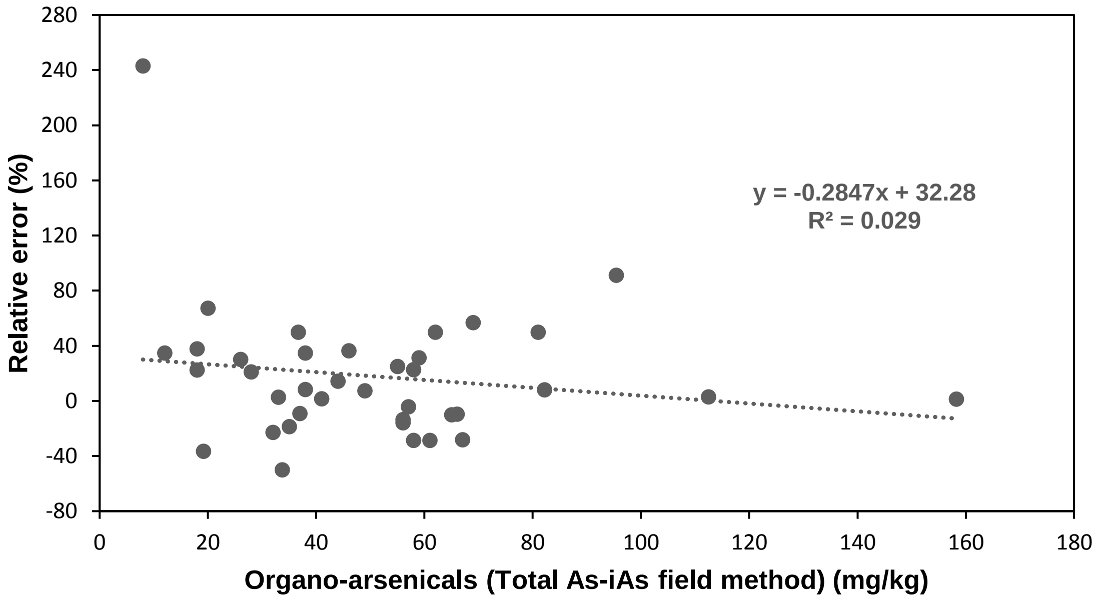

Figure S6 Relative error ((iAs HPLC-ICPMS-iAs Field method)/iAs HPLC-ICPMS)*100 versus the concentration of organo-arsenicals in seaweed samples show no correlation.

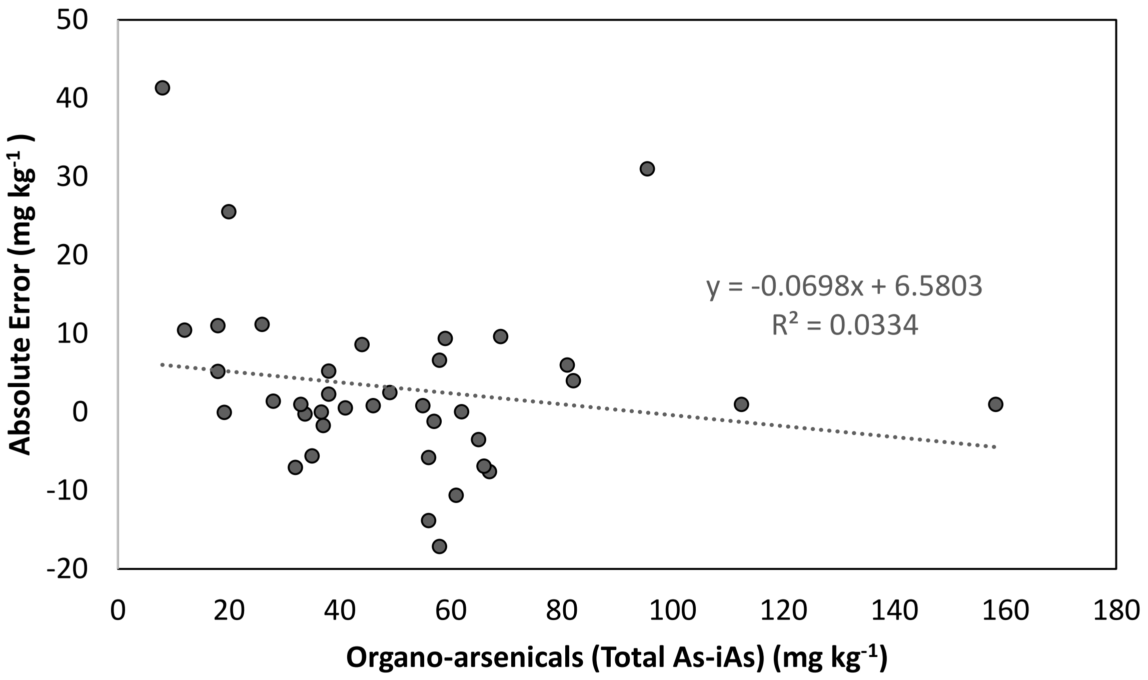

Figure S7 Absolute error (iAs (field method) – iAs (HPLC-ICP-MS)) versus the amount of organo-arsenicals (Total As – iAs (HPLC-ICP-MS)) show no correlation.
